# Supportive care needs of men living with prostate cancer in England: a survey

**DOI:** 10.1038/sj.bjc.6604406

**Published:** 2008-05-27

**Authors:** E Ream, A Quennell, L Fincham, S Faithfull, V Khoo, J Wilson-Barnett, A Richardson

**Affiliations:** 1 King's College London, Division of Health and Social Care Research, Florence Nightingale School of Nursing Midwifery, 5th Floor, Waterloo Bridge Wing, Franklin Wilkins Building, 150 Stamford Street, London SE1 9NN, UK; 2 London Oncology Clinic, 95 Harley Street, London W1G 6AF, UK; 3 Division of Health and Social Care Research, Florence Nightingale School of Nursing Midwifery, 5th Floor, Waterloo Bridge Wing, Franklin Wilkins Building, 150 Stamford Street, London SE1 9NN, UK; 4 European Institute of Health and Medical Sciences, University of Surrey, Stagg Hill, Guildford, Surrey GU2 7TE, UK; 5 The Royal Marsden Hospital NHS Foundation Trust, Fulham Road, London SW3 6JJ, UK; 6 Division of Health and Social Care Research, Florence Nightingale School of Nursing Midwifery, 5th Floor, Waterloo Bridge Wing, Franklin Wilkins Building, 150 Stamford Street, London SE1 9NN, UK; 7 Division of Health and Social Care Research, Florence Nightingale School of Nursing Midwifery, 5th Floor, Waterloo Bridge Wing, Franklin Wilkins Building, 150 Stamford Street, London SE1 9NN, UK

**Keywords:** prostate cancer, lower urinary tract symptoms, quality of life, supportive care need

## Abstract

Men with prostate cancer have various treatment options depending upon their stage of disease, age and presence of comorbidity. However, these treatments typically induce side effects, which generate currently ill-defined supportive care needs. This study examined the supportive care needs of men with prostate cancer within England. A postal questionnaire survey was conducted in six acute NHS Trusts. Seven hundred and forty-one men with prostate cancer participated. They had been diagnosed 3–24 months prior to the survey and had received various treatments. Men surveyed had specific and significant unmet supportive care needs. Areas of greatest need are related to psychological distress, sexuality-related issues and management of enduring lower urinary tract symptoms. High levels of psychological distress were reported, and those reporting psychological distress reported greater unmet supportive care needs. Unmet sexuality-related need was highest in younger men following radical prostatectomy. Lower urinary tract symptoms were almost universal in the sample. Perceived quality of life varied; men unsure of their remission status reported lowest quality of life. Psychological distress impacts significantly on perceived unmet need and is currently not being assessed or managed well in men living with prostate cancer in England.

Prostate cancer is the most common solid tumour malignancy in men. In 2004, 29 406 new cases were diagnosed in England, and comprised 25% of all male cancer diagnoses that year (Office for National Statistics, 2006). Fortunately, the 5-year survival rate for this male cancer is encouraging, reflecting the slow growing nature of the disease in many men and its successful treatment in others. Seventy-one per cent of men are alive 5 years after diagnosis (Office for National Statistics, 2007).

Various treatment options are available; management of men with prostate cancer is generally determined by their stage of disease, age and comorbidity (Harlan *et al*, 2001). Unsurprisingly, men with localised disease have more treatment options, including opportunity for radical treatments including prostatectomy and radiotherapy, than men with locally advanced or metastatic disease. However, there are wide variations in management practices for localised prostate cancer in the United Kingdom, reflecting the lack of consensus over what constitutes best treatment (Hanna *et al*, 2002; Payne and Gillatt, 2007). Different primary treatments for localised treatments typically confer comparative cancer control (Litwin *et al*, 2007). Some urologists and oncologists take account of baseline urological and sexual function in optimising treatment options, usually in discussion with the men themselves. Logically, patient experience will be optimised if the enduring side effects associated with the various treatments, and consequential supportive care needs, are also taken into account.

Supportive care needs can be defined as requirements for care arising during illness and treatment to manage symptoms and side effects, enable adaption and coping, optimise understanding and informed decision-making, and minimise decrements in functioning. Limited evidence, generated primarily by four surveys, exists on the supportive care needs of men living with prostate cancer. One survey explored the supportive care needs of 206 men subscribed to prostate cancer self-help groups in Queensland, Australia (Steginga *et al*, 2001), another investigated the needs of men (*n*=204) with localised prostate cancer in four US sites (Boberg *et al*, 2003), Smith *et al* (2007) conducted a survey in New South Wales, Australia of 978 men under 70 years and newly diagnosed with the disease, whereas Lintz *et al* (2003) conducted the only UK survey. The latter surveyed needs of men attending a non-surgical oncology clinic (*n*=210). These surveys suggest that up to 87% of men with prostate cancer have elements of unmet supportive care need (Steginga *et al*, 2001) with greatest unmet need relating to sexuality and psychological issues (Steginga *et al*, 2001; Lintz *et al*, 2003; Smith *et al*, 2007). Steginga *et al* (2001) and Smith *et al* (2007) determined that high need for patient care and support was associated with lower levels of education. Further, Steginga *et al* (2001) determined that men who had had radiotherapy to the prostate and not in remission were at risk of high unmet need. Unfortunately, evidence generated by these studies is compromised by the size and nature of their samples (none being widely representative of men living with prostate cancer). Further, most surveys were conducted outside the UK and may not translate well to it as health care systems generally, and community care specifically, differs between countries.

To date, the supportive care needs of men with prostate cancer within the United Kingdom have been poorly articulated. To address this, a postal survey was undertaken in England across six sites to determine how the disease and its treatment affect men living with prostate cancer and identify factors that account for this experience.

## SUBJECTS AND METHODS

Data were collected for the survey prior to the conduct of a quasi-experimental study evaluating the role of prostate cancer-specific clinical nurse specialists. These nurse specialists were put in post in various locations across the UK funded by The Prostate Cancer Charity to enhance the care provided to men with this malignancy. The function of the survey was to provide context for the latter evaluation.

The aims of the survey were to:

- Identify unmet supportive care needs in men living with prostate cancer
- Determine predictors of unmet supportive care need

### Study population

A total of 1848 men who had been diagnosed with prostate cancer in the 3–24 months prior to data collection, from across six NHS Trusts, were written to by their consultant urologist inviting them to participate in the study. A standard letter was used. If men did not respond, a further letter was not mailed out. The sites (St George's Healthcare NHS Trust in London, The Hillingdon Hospital NHS Trust, University Hospital Birmingham NHS Foundation Trust, York Hospitals NHS Foundation Trust, Nottingham University Hospitals NHS Trust and Bradford Teaching Hospitals NHS Foundation Trust) were selected to provide two in the north of England, two in the midlands and two in the south. A postal questionnaire was sent to the men (*n*=820) who responded to the invitation. A further questionnaire was posted to non-responders 3 weeks later. There were no exclusion criteria with men of all ages, stages of disease and ethnicity being invited to participate in the survey. Data were collected between November 2004 and September 2005.

### Survey questionnaire

The survey questionnaire comprised four discrete research tools.

1. The Supportive Care Needs Survey (Bonevski *et al*, 2000; Sanson-Fisher *et al*, 2000) is a 34-item questionnaire that encompasses 4 dimensions – Physical and Daily Living (need related to physical symptoms and side effects of treatment), Psychological (need regarding emotions and coping), Sexuality (need relating to sexual relationships), Patient care and Support (need for information on treatment centres and investigations/treatment). Additionally, a seven-item prostate-specific module was used that measures need relating to urinary and bowel function, and self-image. For each item, respondents report the level of need they have from one of the following: no need, need satisfied, low need, moderate need or high need. Validity testing has supported the questionnaire's factor structure; the five domains account for 72% of total variance (McElduff *et al*, 2004). Internal reliability of >0.86 has been consistently reported (Bonevski *et al*, 2000; Steginga *et al*, 2001; McElduff *et al*, 2004). Preceding analysis, data from this tool were classified into ‘no need’ and ‘some need’ according to the author's instructions (McElduff *et al*, 2004). The former category incorporated ‘no need’ and ‘satisfied need’, whereas the latter included ‘low’, ‘moderate’ and ‘high’ need.
2. The International Prostate Symptom Score (IPSS) (Barry *et al*, 1992) was used to determine lower urinary tract symptoms (LUTS). The IPSS is an internationally used, psychometrically sound tool that measures severity of seven aspects: incomplete emptying, frequency, intermittency, urgency, weak stream, straining and nocturia. Respondents record answers on a scale that ranges from 0 (not at all) to 5 (almost always). According to the authors’ instructions, these items were summed to provide an overall score and then categorised to indicate mild (range 0–7), moderate (range 8–19) and severe (range 20–35) symptoms (Barry *et al*, 1992). A further item requires men to respond yes/no regarding whether they are affected by incontinence.
3. The EuroQol EQ-5D and VAS (Visual Analogue Scale) (EuroQol group, 1990) is a widely-used tool to assess respondents’ perceived quality of life. The tool comprises two sections. The first includes five items measuring health state – mobility, self-care, usual activities, pain/discomfort and anxiety/depression. Respondents chose from one of three responses: no problem, some or moderate difficulty, or extreme difficulty. Prior to analysis, data from the EuroQol were transposed to ‘no problems’ and ‘some problems’. The latter incorporated ‘some or moderate difficulty’ and ‘extreme difficulty’. The second section consists of a vertical VAS (0–100) valuing individuals’ health state.
4. A final section comprised additional questions on age and other demographic characteristics, including treatment received, sociodemographic and domestic status, and perceived remission status.

Data were not collected from patient records; demographic and clinical data were attained solely through patient self-report.

### Ethical approval

The research proposal was submitted to and gained MREC approval from the Metropolitan Multicentre Research Ethics Committee (REC reference 04/MRE11/6), prior to conduct of the study.

### Analysis

Demographic and clinical characteristics were described according to the categories of unmet supportive care need. *χ*^2^ tests were undertaken to identify associations. Results reported in this paper relate to demographic and clinical factors that were consistently associated with unmet need. These were age, treatment administered and perceived remission status. Backward logistic regression analysis was performed on The Supportive Care Needs Survey data to determine predictors of need for each domain. Interactions between predictors were explored. The following factors were utilised as predictors: age, educational level, domestic status, mental affect (attained from the EuroQol item on depression/anxiety), time since diagnosis, treatment modality, treatment status, time since treatment and remission status. All analyses were performed using SPSS version 13.0 (SPSS Inc., Chicago, IL, USA).

## RESULTS

Eight hundred and twenty (44%) of the 1847 men informed about the study expressed interest in it and were mailed the survey questionnaire. Of these, 749 returned it (response rate to questionnaire 91%, overall response 41%). Eight questionnaires were incomplete; results are reported on 741 men. Their mean age was 70.2 years (s.d. 8.5). Mean time elapsed since diagnosis was 14.3 months (s.d. 6.2). The sample reported that they had received various treatments following diagnosis, most common were hormone therapy (*n*=376, 51%), radiotherapy to prostate (*n*=209, 28%) and radical prostatectomy (*n*=188, 5%). Some stated that they had had a combination of these therapies. A relatively small proportion reported that they were yet to receive treatment, that is, they were on active monitoring or watchful wait protocols (*n*=124, 17%). Caucasian British males dominated the sample (*n*=675, 91%). Most men had not attained formal educational qualifications (*n*=341, 46%). The majority believed they were in remission (*n*=342, 46%) and at the time data collected were not undergoing any form of treatment for prostate cancer (*n*=344, 47%) (Table 1).

### Supportive care needs

To determine the extent of unmet need within each domain (except the prostate domain), a numeric score was attributed to each category of need (low need scored 1, moderate need scored 2 and high need scored 3) and a mean value calculated. This was not undertaken with the prostate domain as it is relatively new and it is unclear how its individual items relate to one another (McElduff *et al*, 2004). Unmet sexuality-related need was greatest with a mean value of 1.42. All other domains scored on average <1.

The *χ*^2^ tests identified that need related to sexuality was significantly associated with both age (*P*=<0.001) and treatment (*P*=0.001) (Tables 2 and 3). There was a clear downward trend by age (Table 2). With regards to treatment, proportionally more men who had undergone radical prostatectomy reported sexuality-related needs when compared with other subgroups of men (51 *vs* 2–40%). Further 45% of men who had undergone multiple treatments reported some unmet sexuality-related need. Conversely, the relatively small group of men in receipt of brachytherapy experienced little sexuality-related need.

Treatment was also associated with physical and daily living need (*P*<0.001) (Table 3). More men having combinations of treatment or undergoing hormone therapy reported unmet physical and daily living need when compared across treatment regimes. However, it is also worth acknowledging that these treatments are typically prescribed for men with advanced disease. Again, men who had undergone brachytherapy reported fewer needs related to this domain.

Psychological need was associated with remission status (*P*=0.001) as was health system/information need (*P*=<0.001) (Table 4). Psychological need was most prevalent in men who were uncertain of their remission status, whereas health system/information need was most associated with men not in remission.

Data relating to the individual prostate items determined that across three of the individual prostate items (hot flushes, bowel habits and feeling that you have lost part of your manhood), need was significantly associated with treatment type (*P*⩽0.001). No one treatment was more greatly associated with all prostate symptoms (Table 5). Rather, hot flushes were most problematic in men having hormone therapy, bowel problems with brachytherapy and multiple treatments, and feeling that you have lost part of your manhood most associated with radical prostatectomy and multiple treatment combinations.

### Predictors of unmet supportive care need

Logistic regression revealed that treatment, age and mental affect were significant factors impacting on the Physical and Daily living domain (Table 6). Older men, and men who received multiple treatments, were more likely to express unmet physical and daily living need. However, the factor that placed men most at risk of need in this domain was low mental affect. Men with some depression and/or anxiety were five times more likely to express outstanding physical and daily living need compared with men not reporting anxiety and/or depression. With regard to unmet psychological need, two predictors were identified – time since diagnosis and, unsurprisingly, low mental affect. Psychological need was less likely to arise as time elapsed following treatment, but was 10 times more likely in men with low mood. Predictors of sexuality-related need comprised age, treatment, treatment status and mental affect. Prostatectomy and hormone therapy were the two treatments that placed men at risk of sexuality-related need. However, the single most important factor was once again mental affect. Low mood increased risk of sexuality-related need by 3.7 times. As with previous elements of supportive care need, low mood was the most significant predictor of both the Patient Care and Support domain and Health Systems/Information domain.

### Experience of LUTS

In the month prior to the survey, almost the entire sample (*n*=726, 97%) experienced at least one of the following: incomplete emptying, frequency, intermittency, urgency, weak stream, straining or nocturia. Responses to individual items were summed and classified to indicate whether overall urinary symptoms were mild (scores 0–7), moderate (8–19) or severe (20–35). This revealed that 356 men (50%) were mildly symptomatic, 288 men (39%) moderately so and 82 men (11%) had severe urinary symptoms. Of the 726 men who completed the final item regarding incontinence, 144 (19%) reported difficulty with leaking urine.

Inferential statistics confirmed that perceived severity of urinary symptoms was impacted by treatment (*P*<0.001), remission status (*P*<0.001) and time since last treatment (*P*=0.029). Urinary symptoms were least severe in men who were in remission, and/or had undergone radical prostatectomy, and/or completed their treatment regimen 19–24 months prior to the survey.

### Quality of life

Around a quarter of the sample experienced problems with four of the five quality of life domains measured. Moderate or extreme anxiety or depression was reported by 227 (30%) men, difficulty with undertaking usual activities was reported by a further 221 men (30%), some or extreme pain was reported by 192 men (26%), and problems with mobility arose in 167 (22%) of those sampled. Conversely, difficulty with self-care was evident in only 56 men (7%).

The *χ*^2^ testing identified that age, treatment and remission status were significantly associated with impairments to these dimensions of quality of life (Tables 2, 3 and 4). Age was significantly associated with reduced mobility (*P*<0.001) and difficulty in performing usual activities (*P*<0.001), with older men reporting more problems with these domains (Table 2). Treatment was significantly associated with both problems with mobility (*P*<0.001) and pain (*P*<0.001). Certain treatment regimes – hormone therapy, watchful waiting/active monitoring and those entailing multiple treatments – were allied with greater pain and reduced mobility (Table 3). Remission status was associated with four quality of life domains: depression/anxiety (*P*<0.001), reduced mobility (*P*<0.001), difficulty performing usual activities (*P*<0.001) and pain (*P*=0.001) (Table 4). Across each of these four domains, proportionally more men not in remission reported some difficulty when compared with those either in remission or unsure of their remission status. Further men unsure of their remission status appeared to report more problems with these areas of quality of life compared with men in remission.

## DISCUSSION

This survey of supportive care need determined that many men living with prostate cancer have areas of high unmet need. This was particularly evident with regards to psychological care needs. The survey identified high levels of psychological distress within the sample even though the majority (57%) had completed treatment over a year previously. Need for psychological care was particularly high in men not in remission and in those uncertain of their remission status. This might be due to heightened feelings of uncertainty in these men. Recognition of the contribution of uncertainty to psychological distress in men with prostate cancer is not new. It is acknowledged to be particularly associated with watchful wait or active monitoring protocols (Wallace, 2003; Bailey *et al*, 2004) and reported to be present in survivors of the disease (Talcott, 2006).

Unfortunately, this study did not explicitly survey the prevalence of either anxiety or depression. Indeed, there is little evidence on how much these psychological symptoms affect men treated for prostate cancer. Some understanding is provided by Korfage *et al* (2006) who undertook a 5-year follow-up in Rotterdam of 299 men treated for local disease. They determined that 25% men experienced high anxiety before treatment. This decreased 6 months post-treatment and remained lower throughout the 5-year follow-up period. They concluded that most, but not all, men adjust well psychologically following treatment for localised prostate cancer. The current study suggests that men unsure of their remission status may not adjust as well. This supports the National Audit Office findings (National Audit Office, 2005) that many men with prostate cancer wish more information on how their disease has responded to treatment.

The current study identified that psychological need typically decreased with time, as did Steginga *et al* (2004). The latter determined that at diagnosis 63% of men had high decision-related distress that persisted for 42% of men 12 months after treatment.

The need for systematic assessment and better management of psychological distress within men with prostate cancer was evident in this study. Regression analysis identified that this variable was a strong predictor of supportive care need. Across all domains measured, men with psychological distress had greater unmet supportive care needs. Nurse-led interventions for uncertainty have been developed and evaluated (Mishel *et al*, 2002; Bailey *et al*, 2004), as have those for depression (McCorkle *et al*, 2007). Arguably, a clinical nurse specialist could deliver such interventions following the requisite training.

Additionally, a high level of unmet sexuality-related need was reported. Younger men, with a good prognosis following curative radical prostatectomy, were more greatly affected. Radical prostatectomy is known to impact on libido and erectile function/sexual performance (Talcott *et al*, 2003; Matthew *et al*, 2005; Ponholzer *et al*, 2006). However, the men in this group are also more likely to be sexually active due to their age, and so find impotency and changes in sexual desire more problematic.

As with uncertainty and psychological distress, problems with sexuality are not necessarily evident; men may be reluctant to bring sexuality-related issues to the fore, given the stigma associated with them. Clinically important sexuality-related need will only be identified through systematic assessment.

This survey also identified that LUTS were almost universal in men up to 2 years following treatment for prostate cancer. Lower urinary tract symptoms transcend stage of disease, treatment administered and stage of treatment. Their high prevalence in this survey (97%) is indicative of the difficulty associated with resolving them. However, prevalence is not dissimilar to that within a general population of elderly men (>65 years) without prostate cancer (Taylor *et al*, 2006). Yet, although men in the general population report a similar presence of LUTS, they appear less severe (Taylor *et al*, 2006). Fifty per cent of men sampled in this survey of men living with prostate reported these symptoms to be moderate or severe. This would appear unsatisfactory and may reflect current service delivery. Services for incontinence are well established within health care. Specific services for LUTS, however, are less common. The high prevalence and severity of LUTS in this sample would suggest that these symptoms require increased clinical attention within prostate services. They should be addressed from presentation and diagnosis, through treatment and during follow-up.

However, although this is the largest survey of its kind to date to be conducted in the UK and to have enjoyed a relatively healthy response rate, its limitations must be considered. Only men who conveyed interest in the study to the research team were invited to participate. This opt-in approach was stipulated by the approving ethics committee in line with current requirement and illustrates the impact of it on sample selection (Hewison and Haines, 2006). The sample may be unrepresentative of all men living with this disease. Men from black and mixed ethnic (BME) groups were poorly represented even though two research sites were selected for their high proportion of BME groups in their local populations. The study was also of cross-sectional design and included men at different points in the treatment and recovery pathway. Many men had completed treatment. Further, as baseline measures of supportive care need, symptoms and quality of life prior to treatment were not attained, it is not possible to determine how much the differences in outcome reported between treatment groups relates to baseline factors rather than treatment-related ones. Additionally, the survey relied on self-reported data related to treatment received and remission status. This approach is subject to reporting bias.

However, the study raises important points for debate concerning the current provision of services for men with prostate cancer in England. It strongly indicates that further research is necessary to provide robust evidence on the long-term impact of prostate cancer. From a practice perspective, it suggests that greater attention should be paid to symptoms and problems that men may be reticent to raise with professionals, perhaps due to perceived stigma associated with them. These include sexuality-related problems, psychological distress, incontinence and LUTS, and altered bowel functioning. Management of these problems would be improved through routine systematic screening of men at risk of developing them – this survey and others have determined that particular treatments are strongly associated with particular sequelae. Further, resources need to be devoted to ensuring that all men have access to the relevant specialist services, some of which could be provided by specialist nurses with the requisite training. Finally, this survey has highlighted that psychological distress is currently not being assessed or managed well in men living with prostate cancer in England and this contributes to much of their perceived unmet supportive care need.

## Figures and Tables

**Table 1:** Characteristics of the sample (*N*=741)

	***N***	**%**
*Age (years)*		
<65	180	24
65–69	164	22
70–75	171	23
75–80	121	16
80<	105	14
		
*Time since diagnosis (months)*		
3–6	96	13
7–12	223	30
13–18	204	28
19–24	218	29
		
*Treatment received* a		
Hormone therapy	376	51
Radiotherapy to prostate	209	28
Radical prostatectomy	188	25
Watchful wait	124	17
Brachytherapy	26	4
Other treatments	54	7
		
*State of remission*		
In remission	342	46
Not in remission	231	32
Uncertain	142	19
Missing data	26	3
		
*Education*		
No formal qualifications	341	46
GCSE (or equivalent)	110	15
A level (or equivalent)	95	13
Higher educational qualification	131	18
Missing data	64	9
		
*Ethnicity*		
British	675	91
Irish	22	3
African or Caribbean	22	3
Indian, Pakistani or other Asian background	8	1
Mixed race	14	2
		
*Treatment status*		
Treatment in past	344	47
On treatment	273	37
Watchful wait/no treatment	122	17
Missing data	2	—

a Some individuals received more than one type of therapy.

**Table 2:** Significant associations between age and supportive care need and quality of life domains

	**Age groups**					
	**<65 years**	**65–69 years**	**70–74 years**	**75–80 years**	**>80 years**	
	***N*** **(%)**	***N* (%)**	***N* (%)**	***N* (%)**	***N* (%)**	***P*-value**
*Sexuality*						
Some need	93 (52)	74 (45)	60 (35)	40 (33)	27 (20)	<0.001
						
*Mobility*						
Some mobility problems	28 (16)	29 (18)	30 (18)	38 (31)	42 (40)	<0.001
						
*Usual activities*						
Some problems with usual activities	48 (27)	39 (23)	52 (30)	37 (31)	46 (44)	0.005

**Table 3:** Significant associations between treatment and supportive care need and quality of life domains

	**Treatment**							
	**Watchful wait/active monitoring**	**Radiotherapy to prostate**	**Radiotherapy to prostate+hormone therapy**	**Hormone therapy**	**Radical prostatectomy**	**Multiple combinations**	**Brachy therapy**	
	***N* (%)**	***N* (%)**	***N* (%)**	***N* (%)**	***N* (%)**	***N* (%)**	***N* (%)**	***P*-value**
*Sexuality*								
Some need	31 (26)	14 (33)	57 (40)	67 (35)	82 (51)	36 (45)	11 (2)	0.001
								
*Physical and daily living*								
Some physical and daily living need	34 (29)	16 (37)	60 (43)	77 (42)	39 (25)	35 (45)	6 (1)	<0.001
								
*Mobility*								
Some mobility problems	35(29)	5 (12)	34 (24)	61 (32)	15 (9)	15 (29)	1 (4)	<0.001
								
*Pain*								
Some pain/Discomfort	32 (27)	11 (26)	47 (33)	53 (27)	20 (12)	22 (43)	6 (23)	<0.001

**Table 4:** Significant associations between remission status and supportive care need and quality of life domains

	**In remission**			
	**No**	**Yes**	**Unsure**	
	***N* (%)**	***N* (%)**	***N* (%)**	***P*-value**
*Health system/information*				
Some health system/information need	118 (51)	112 (33)	59 (43)	<0.001
				
*Psychology*				
Some psychological need	63 (27)	72 (21)	43 (31)	0.001
				
*Depression/anxiety*				
Some depression and/or anxiety	91 (40)	83 (24)	48 (34)	<0.001
				
*Mobility*				
Some mobility problems	70 (30)	58 (17)	35 (25)	0.001
				
*Usual activities*				
Some problems with usual activities	86 (37)	80 (23)	47 (33)	0.001
				
*Pain*				
Some pain/discomfort	88 (38)	66 (19)	32 (23)	0.001

**Table 5:** Significant associations between treatment and prostate-specific supportive care need items

	**Treatment**							
	**Watchful wait/active monitoring**	**Radiotherapy to prostate**	**Radiotherapy to prostate+hormone therapy**	**Hormone therapy**	**Radical prostatectomy**	**Multiple combinations**	**Brachytherapy**	
	***N* (%)**	***N* (%)**	***N* (%)**	***N* (%)**	***N* (%)**	***N* (%)**	***N* (%)**	***P*-value**
*Hot flushes*								
Some need	11 (9)	7 (16)	42 (30)	61 (32)	8 (5)	15 (29)	2 (8)	<0.001
								
*Bowel problems*								
Some need	23 (19)	7 (16)	35 (25)	38 (20)	13 (8)	18 (35)	10 (38)	<0.001
								
*Lost part of manhood*								
Some need	28 (23)	11 (26)	58 (41)	60 (31)	73 (45)	22 (42)	7 (27)	0.001

**Table 6:** Predictors of unmet supportive care need

**Domain**	**Predictor**	**Odds ratio**	**95% CI**	***P*-value**
Physical and daily living	Depression	5.9	4.1, 8.6	<0.001
	*Treatment*			0.003
	Radical prostatectomy	1.1	0.6, 2.1	0.694
	Radiotherapy to prostate	2.3	1.0, 5.2	0.045
	Radiotherapy to prostate+hormone therapy	2.5	1.4, 4.4	0.002
	Hormone therapy	1.9	1.1, 3.2	0.025
	Brachytherapy	1.0	0.3, 3.0	0.996
	Multiple treatments	3.5	1.6, 7.6	0.002
	Watchful wait/active monitoring	—	—	—
	Age	1.0	1.0, 1.1	0.02
				
Psychological	Time since diagnosis	1.0	0.9, 1.0	0.047
	Depression	10.7	7.0, 16.2	<0.001
				
Sexuality	Age	1	0.9, 1.0	<0.001
	Depression	3.7	2.6, 5.2	<0.001
	*Treatment*			0.009
	Radical prostatectomy	2.7	1.5, 4.8	0.001
	Radiotherapy to prostate	1.5	0.6, 3.3	0.378
	Radiotherapy to prostate and hormone therapy	2.4	1.3, 4.4	0.005
	Hormone therapy	3.4	1.7, 7.1	0.001
	Brachytherapy	2.4	0.9, 6.1	0.076
	Multiple treatments	2.9	1.3, 6.5	0.012
	Watchful wait/active monitoring	—	—	—
				
	*Treatment status*			0.005
	On treatment	0.5	0.3, 0.8	0.005
	Treatment in past	—	—	—
				
Patient care and support	Age	1.0	0.9, 1.0	0.023
	Depression	5.7	3.9, 8.2	<0.001
				
Health systems/information	Depression	3.8	2.7, 5.3	<0.001
	*Remission status*			0.001
	In remission	0.5	0.4, 0.7	<0.001
	Do not know	0.8	0.5, 1.2	0.232
	Not in remission	—	—	—
